# Supplementary material for: Reconciling periodic rhythms of large-scale biological networks by optimal control
Source: R Soc Open Sci. 2020 Jan 8;7(1):191698. doi: 10.1098/rsos.191698 (PMC7029949; doi:10.1098/rsos.191698)
Supplement: Parameters setting in the optimization problems [file rsos191698supp1.docx]

**Reconciling periodic rhythms of large-scale biological networks by optimal control**

Supplementary Data 1

**Table S1. Parameters in the optimization problems of three case studies**

| Case studies | Scenarios | *N* | *M* | *t_f_* | *α* | *β* | *γ* | *u*^min^ | *u*^max^ |
| --- | --- | --- | --- | --- | --- | --- | --- | --- | --- |
| Chaotic  system | All | 3 | 3 | 80 | 1 | 1 | 1 | 0.1 | 6 |
| Mammalian circadian network | CYA2 to CYA1 | 21 | 21 | 90 | 1 | 1 | 1 | 0.1 | 5 |
|  | 6h time-lag | 21 | 21 | 120 | 1 | 1 | 50 | 0.1 | 5 |
|  | 12h time-lag | 21 | 21 | 120 | 1 | 1 | 1 | 0.1 | 1.5 |
| Gastric cancer network | POA6 to CYA | 48 | 48 | 90 | 1 | 1 | 2 | 0.1 | 3 |
|  | POA6 to POA1 | 48 | 48 | 50 | 10 | 10 | 1 | 0.1 | 3 |

It should be noted that the values of weighting factors *α*, *β*, and *γ* are selected to ensure the optimization solutions and the boundary values should be taken from experiments or data from clinical trials. The values used in the three case studies in this paper are only for the demonstration of our results in the system level.
