# Supplementary material for: Reconciling periodic rhythms of large-scale biological networks by optimal control
Source: R Soc Open Sci. 2020 Jan 8;7(1):191698. doi: 10.1098/rsos.191698 (PMC7029949; doi:10.1098/rsos.191698)
Supplement: Optimal results of chaotic system [file rsos191698supp2.docx]

**Reconciling periodic rhythms of large-scale biological networks by optimal control**

Supplementary Data 2

1. **Optimal results in chaotic system**

According to the results obtained by the optimization approach, different state transitions are realized between point attractor (POA), cyclic attractor (CYA) and chaotic attractor (CHA) as shown in Figure S1. Herein, we chose the state transition from CYA to CHA, from CYA to POA, from POA to CYA and the transition from POA to CHA to demonstrate trajectories of the state variables and the time tracks of the required control variables.


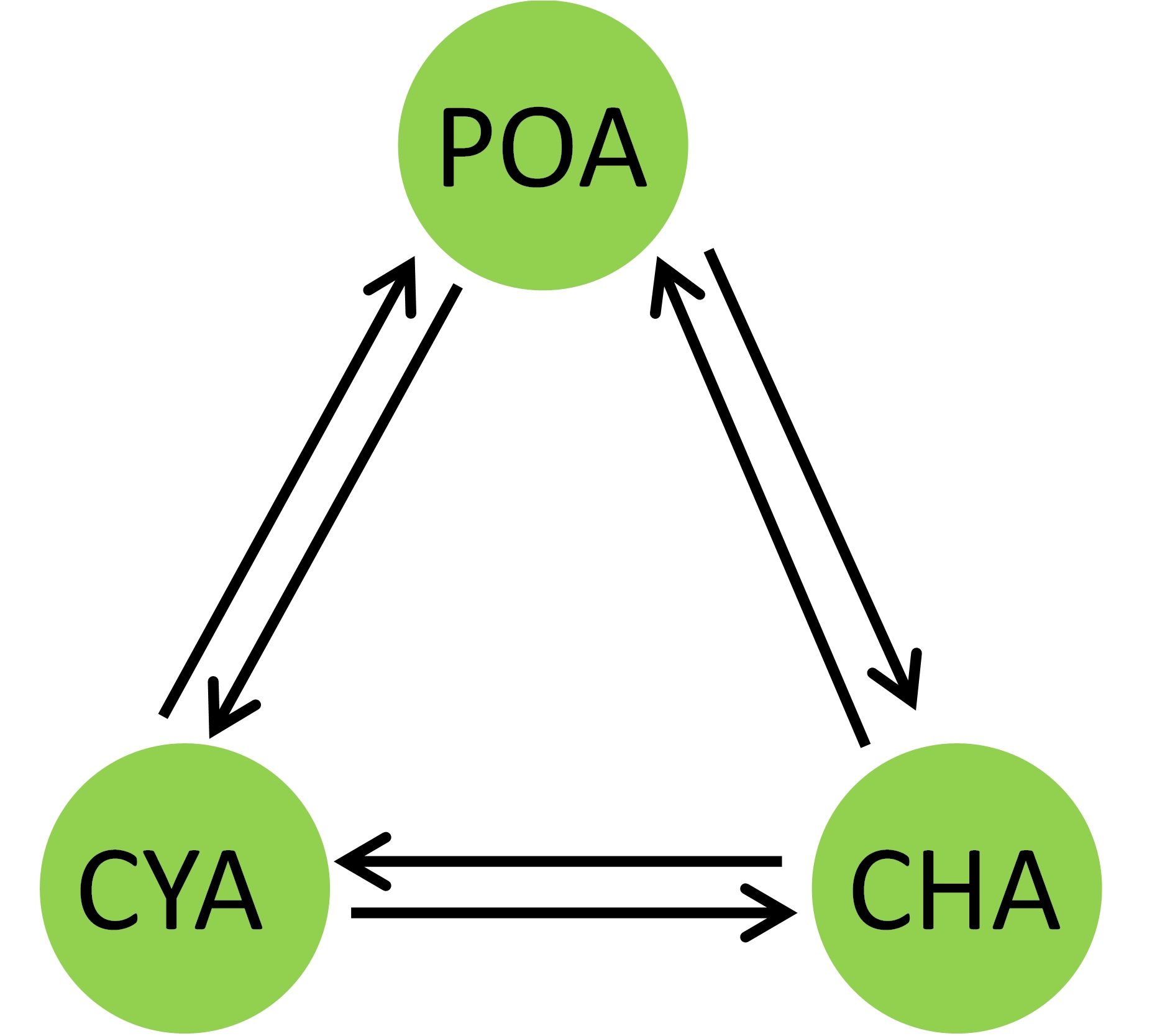


Figure S1 State transitions of the chaotic system. The green circles represent the attractors; the arrow line describes the state transition between different attractors.

The numerical results of state transition from CYA to CHA, from CYA to POA, from POA to CYA and the transition from POA to CHA are shown in Figure S2, Figure S3, Figure S4, Figure S5, respectively. In these figures x1, x2, and x3 are time tracks of the state variables during the optimization process; x1des, x2des and x3des are trajectories of desired attractor. In all these state transitions, the system starts to be controlled at the same time *t*=32, however, the time when the system reaches the steady state and converges to the desired attractor is different. For example at time *t*=47.2, CYA is driven into CHA as shown in Figure S2, while at time *t*=44.8 the attractor CYA is steered into attractor POA as illustrated in Figure S3. Also, the control profiles identified by our optimization algorithm are different for different state transitions. For instance, in the state transition from POA to CYA, the maximal value of *u*_3_ is 3.815 as shown in Figure S4B; however, the maximal value in the state transition from POA to CHA is 2.888 in Figure S5B.

The corresponding control variables and their profiles for the four different state transitions are shown in Figure S2B, Figure S3B, Figure S4B and Figure S5B, respectively. The minimum control variable set N_CON_ for performing the desired state transition is equal to 1, which means that one single decision variable is enough for these state transitions. Despite that *u*_3_ is identified as the control variable in these transitions, they have different control profiles as mentioned above, where the initial value is 1, and it is back to original value after the control target is reached.


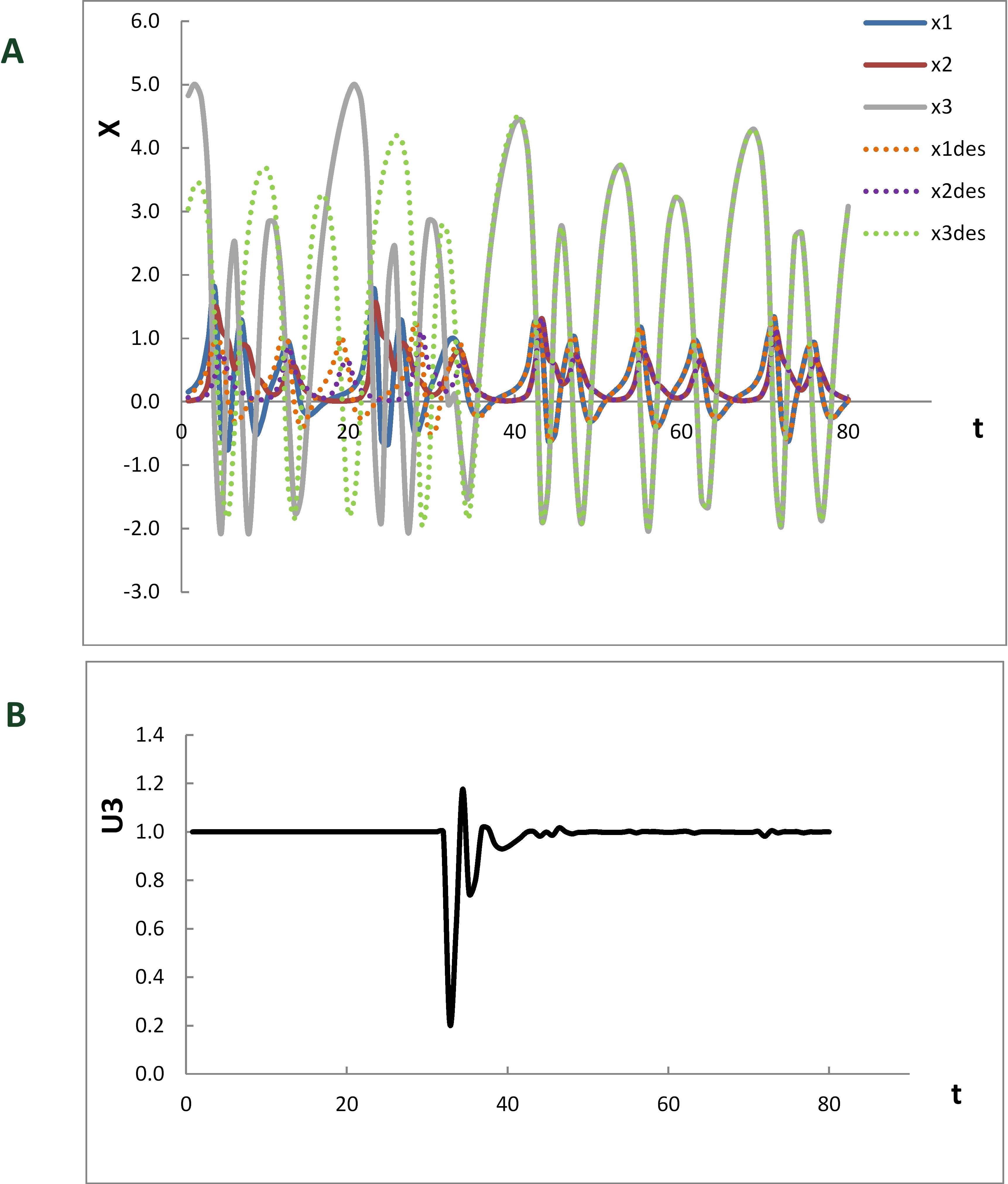


Figure S2 Results of the state transition from CYA to CHA of the chaotic system. (A)Trajectories of x1, x2, x3, x1des, x2des and x3des. (B) The profile of regulatory variable u3.


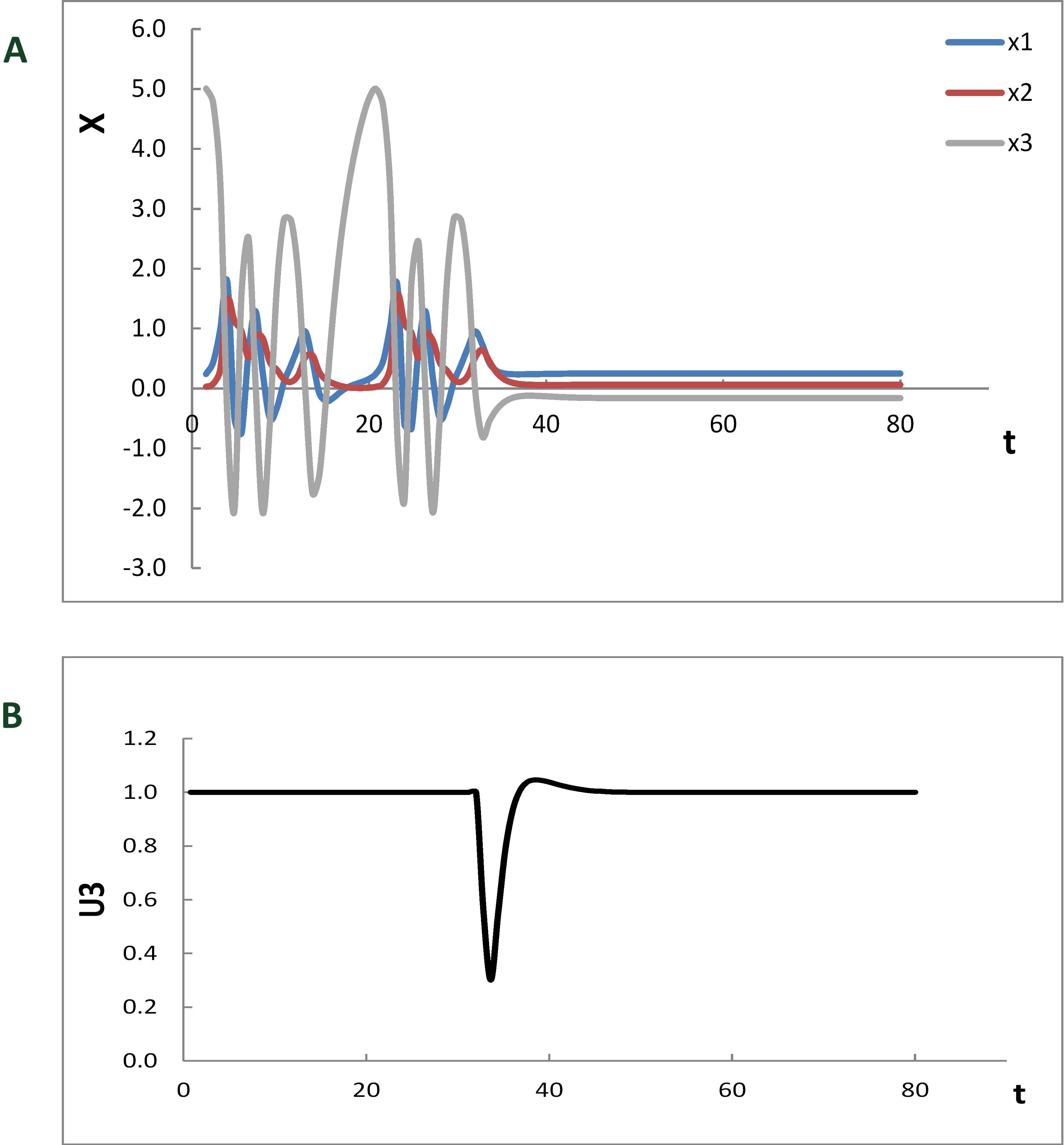


Figure S3 Results of the state transition from CYA to POA of the chaotic system. (A) Time tracks of x1, x2 and x3. (B) The profile of regulatory variable u3.


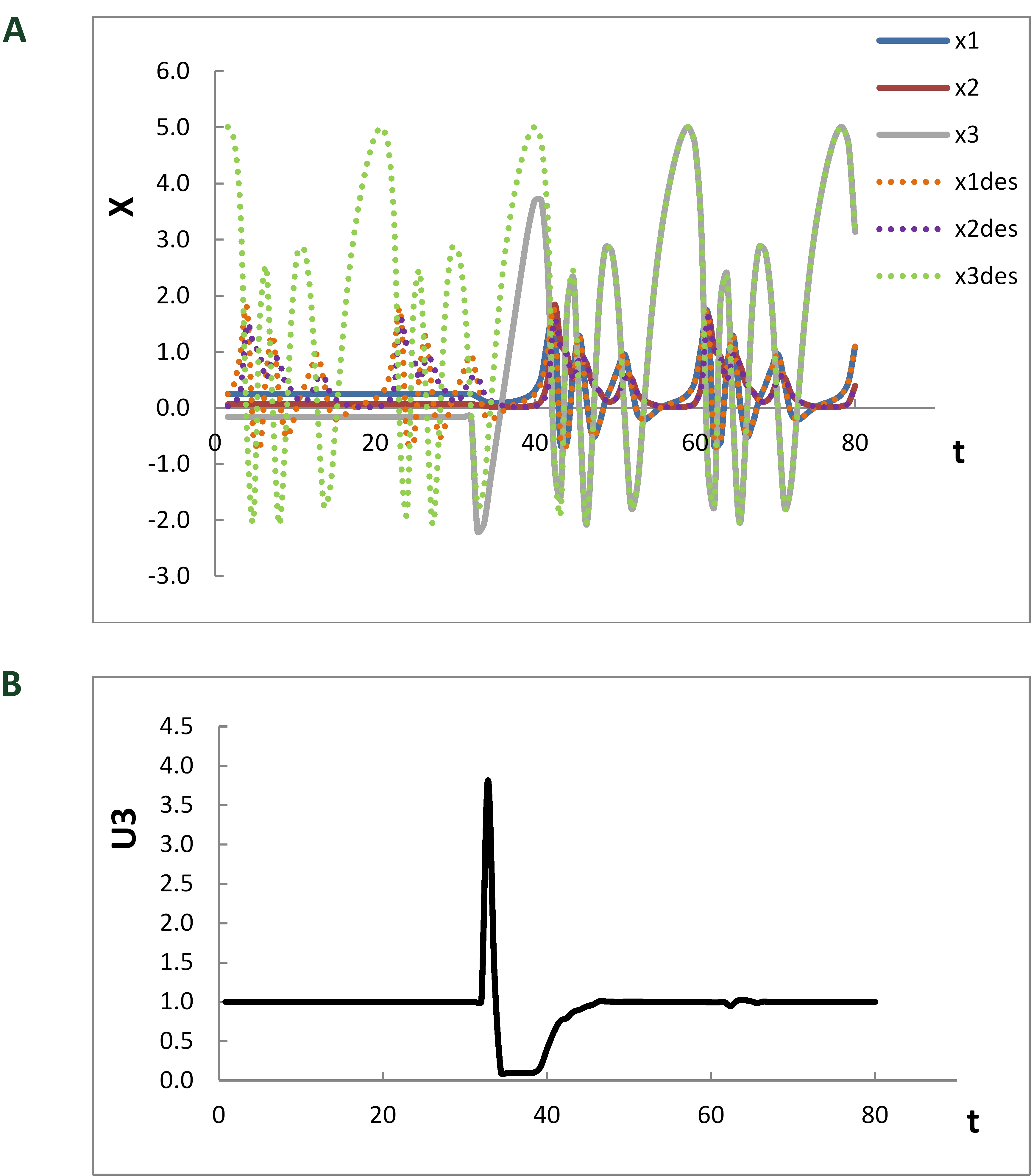


Figure S4 Results of the state transition from POA to CYA of the chaotic system. (A) Trajectories of x1, x2, x3, x1des, x2des and x3des. (b) The profile of regulatory variable u3.

**
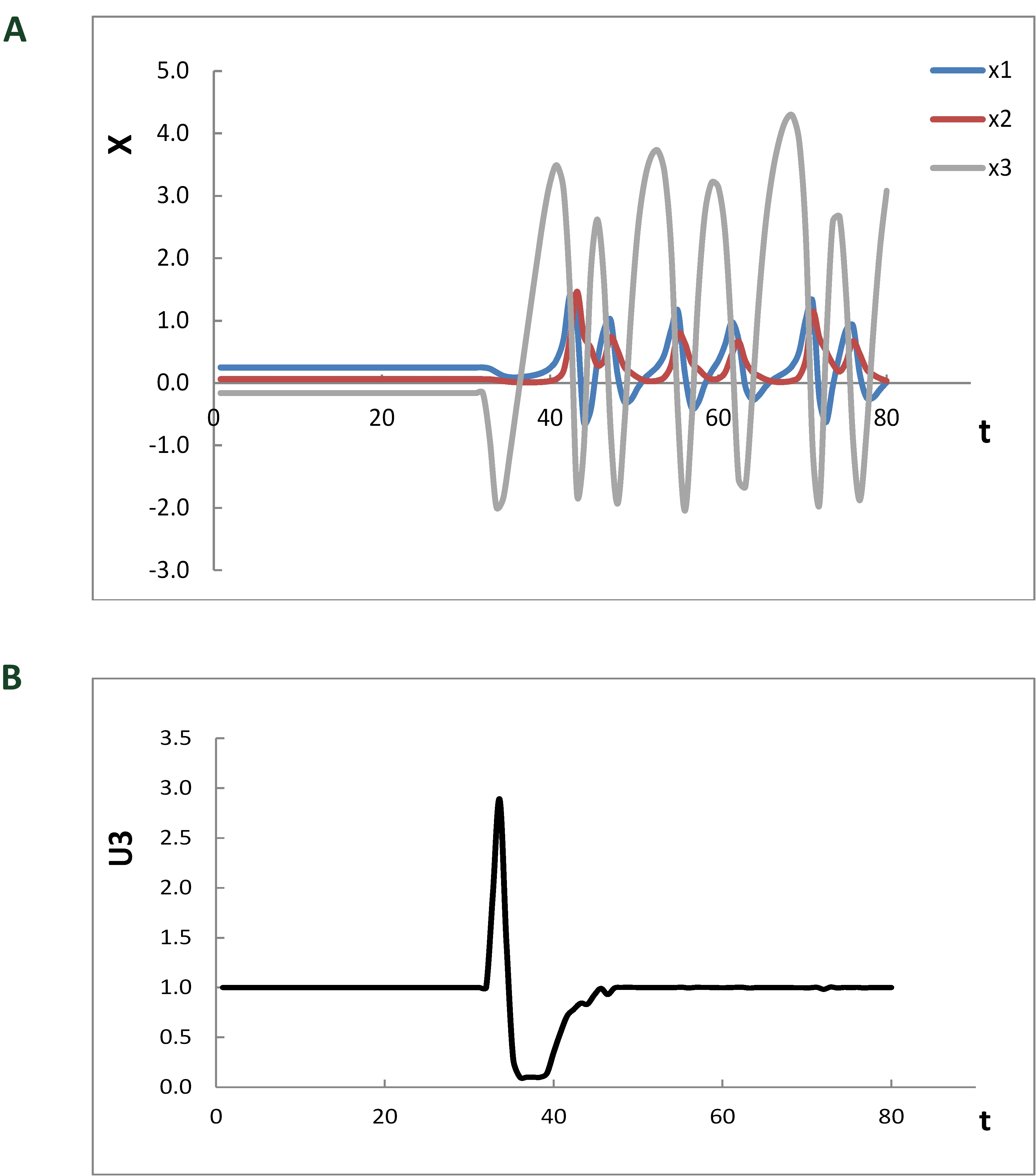
**

Figure S5 Results of the state transition from POA to CHA of the chaotic system. (A) Time tracks of x1, x2 and x3. (B) The profile of regulatory variable u3.

1. **Different guess values for robust problem**

**Table S2. Different guess values of state variables and control variables in the optimization program for the state transition from CHA to POA.**

|  | $x_{1}^{guess}$ | $x_{2}^{guess}$ | $x_{3}^{guess}$ | $k_{1}^{guess}$ | $k_{2}^{guess}$ | $k_{3}^{guess}$ |
| --- | --- | --- | --- | --- | --- | --- |
| 1 | -1.4 | 0.1 | -2.9 | 0.1 | 0.1 | 0.1 |
| 2 | -1.1 | 0.3 | -2.5 | 6.0 | 6.0 | 6.0 |
| 3 | 2.4 | 1.8 | 5.9 | 0.2 | 0.2 | 0.2 |
| 4 | 2.3 | 1.5 | 5.5 | 5.0 | 5.0 | 5.0 |
| 5 | 0.25 | 0.06 | -0.16 | 1.0 | 1.0 | 1.0 |
| 6 | 0 | 0 | 0 | 0.5 | 0.5 | 0.5 |
| 7 | 0.5 | 1.0 | 1.5 | 3 | 3 | 3 |
| 8 | 0.6 | 1.2 | 2.5 | 0.5 | 3.0 | 2.7 |
| 9 | 2.0 | 0.2 | 4.3 | 5.0 | 0.8 | 3.6 |
| 10 | -1.0 | 0.7 | 4.0 | 3.0 | 2.1 | 4.5 |
