# Supplementary material for: Reconciling periodic rhythms of large-scale biological networks by optimal control
Source: R Soc Open Sci. 2020 Jan 8;7(1):191698. doi: 10.1098/rsos.191698 (PMC7029949; doi:10.1098/rsos.191698)
Supplement: Model equations for biological networks [file rsos191698supp3.docx]

**Reconciling periodic rhythms of large-scale biological networks by optimal control**

Supplementary Data 3

**1. Model equations for mammalian circadian rhythm network:**

$$\frac{dx_{1}}{dt}=u_{1}*\left( V_{0,Per1}+V_{1,Per1}\frac{x_{21}^{n_{a1,Per1}}}{\left( {KA}_{1,Per1}^{n_{a1,Per1}}+x_{21}^{n_{a1,Per1}} \right)} \right)*\frac{{KI}_{1,Per1}^{n_{i1,Per1}}}{\left( {KI}_{1,Per1}^{n_{i1,Per1}}+x_{17}^{n_{i1,Per1}} \right)}*\frac{{KI}_{2,Per1}^{n_{i2,Per1}}}{\left( {KI}_{2,Per1}^{n_{i2,Per1}}+{x_{19}}^{n_{i2,Per1}} \right)}*\frac{{KI}_{3,Per1}^{n_{i3,Per1}}}{\left( {KI}_{3,Per1}^{n_{i3,Per1}}+x_{18}^{n_{i3,Per1}} \right)}*\frac{{KI}_{4,Per1}^{n_{i4,Per1}}}{\left( {KI}_{4,Per1}^{n_{i4,Per1}}+x_{20}^{n_{i4,Per1}} \right)}-k_{m,Per1}x_{1}$$

$$\frac{dx_{2}}{dt}=u_{2}*(V_{0,Per2}+V_{1,Per2}\frac{x_{21}^{n_{a1,Per2}}}{\left( {KA}_{1,Per2}^{n_{a1,Per2}}+x_{21}^{n_{a1,Per2}} \right)}*\frac{{KI}_{1,Per2}^{n_{i1,Per2}}}{\left( {KI}_{1,Per2}^{n_{i1,Per2}}+{x_{17}}^{n_{i1,Per2}} \right)}*\frac{{KI}_{2,Per2}^{n_{i2,Per2}}}{\left( {KI}_{2,Per2}^{n_{i2,Per2}}+{x_{19}}^{n_{i2,Per2}} \right)}*\frac{{KI}_{3,Per2}^{n_{i3,Per2}}}{\left( {KI}_{3,Per2}^{n_{i3,Per2}}+x_{18}^{n_{i3,Per2}} \right)}* \frac{{KI}_{4,Per2}^{n_{i4,Per2}}}{\left( {KI}_{4,Per2}^{n_{i4,Per2}}+x_{20}^{n_{i4,Per2}} \right)}-k_{m,Per2}x_{2}$$

$$\frac{dx_{3}}{dt}=u_{3}*\left( V_{0,Cry1}+V_{1,Cry1}\frac{x_{21}^{n_{a1,Cry1}}}{\left( {KA}_{1,Cry1}^{n_{a1,Cry1}}+x_{21}^{n_{a1,Cry1}} \right)}+V_{2,Cry1}\frac{x_{16}^{n_{a2,Cry1}}}{\left( {KA}_{2,Cry1}^{n_{a2,Cry1}}+x_{16}^{n_{a2,Cry1}} \right)} \right)*\frac{{KI}_{1,Cry1}^{n_{i1,Cry1}}}{\left( {KI}_{1,Cry1}^{n_{i1,Cry1}}+{x_{17}}^{n_{i1,Cry1}} \right)}*\frac{{KI}_{2,Cry1}^{n_{i2,Cry1}}}{\left( {KI}_{2,Cry1}^{n_{i2,Cry1}}+{x_{19}}^{n_{i2,Cry1}} \right)}*\frac{{KI}_{3,Cry1}^{n_{i3,Cry1}}}{\left( {KI}_{3,Cry1}^{n_{i3,Cry1}}+x_{18}^{n_{i3,Cry1}} \right)}*\frac{{KI}_{4,Cry1}^{n_{i4,Cry1}}}{\left( {KI}_{4,Cry1}^{n_{i4,Cry1}}+x_{20}^{n_{i4,Cry1}} \right)}*\frac{{KI}_{5,Cry1}^{n_{i5,Cry1}}}{\left( {KI}_{5,Cry1}^{n_{i5,Cry1}}+x_{13}^{n_{i5,Cry1}} \right)}-k_{m,Cry1}x_{3}$$

$$\frac{dx_{4}}{dt}=u_{4}*\left( V_{0,Cry2}+V_{1,Cry2}\frac{x_{21}^{n_{a1,Cry2}}}{\left( {KA}_{1,Cry2}^{n_{a1,Cry2}}+x_{21}^{n_{a1,Cry2}} \right)}+V_{2,Cry2}\frac{x_{16}^{n_{a2,Cry2}}}{\left( {KA}_{2,Cry2}^{n_{a2,Cry2}}+x_{16}^{n_{a2,Cry2}} \right)} \right)*\frac{{KI}_{1,Cry2}^{n_{i1,Cry2}}}{\left( {KI}_{1,Cry2}^{n_{i1,Cry2}}+{x_{17}}^{n_{i1,Cry2}} \right)}*\frac{{KI}_{2,Cry2}^{n_{i2,Cry2}}}{\left( {KI}_{2,Cry2}^{n_{i2,Cry2}}+{x_{19}}^{n_{i2,Cry2}} \right)}*\frac{{KI}_{3,Cry2}^{n_{i3,Cry2}}}{\left( {KI}_{3,Cry2}^{n_{i3,Cry2}}+x_{18}^{n_{i3,Cry2}} \right)}*\frac{{KI}_{4,Cry2}^{n_{i4,Cry2}}}{\left( {KI}_{4,Cry2}^{n_{i4,Cry2}}+x_{20}^{n_{i4,Cry2}} \right)}*\frac{{KI}_{5,Cry2}^{n_{i5,Cry2}}}{\left( {KI}_{5,Cry2}^{n_{i5,Cry2}}+x_{13}^{n_{i5,Cry2}} \right)}-k_{m,Cry2}x_{4}$$

$$\frac{dx_{5}}{dt}={u_{5}*V}_{1,Rev-erb\alpha}\frac{x_{21}^{n_{a1,Rev-erb\alpha}}}{({KA}_{1,Rev-erb\alpha}^{n_{a1,Rev-erb\alpha}}+x_{21}^{n_{a1,Rev-erb\alpha}})}*\frac{{KI}_{1,Rev-eb\alpha}^{n_{i1,Rev-eb\alpha}}}{\left( {KI}_{1,Rev-eb\alpha}^{n_{i1,Rev-eb\alpha}}+x_{17}^{n_{i1,Rev-eb\alpha}} \right)}*\frac{{KI}_{2,Rev-erb\alpha}^{n_{i2,Rev-erb\alpha}}}{\left( {KI}_{2,Rev-erb\alpha}^{n_{i2,Rev-erb\alpha}}+x_{19}^{n_{i2,Rev-erb\alpha}} \right)}*\frac{{KI}_{3,Rev-erb\alpha}^{n_{i3,Rev-erb\alpha}}}{\left( {KI}_{3,Rev-erb\alpha}^{n_{i3,Rev-erb\alpha}}+x_{18}^{n_{i3,Rev-erb\alpha}} \right)}*\frac{{KI}_{4,Rev-erb\alpha}^{n_{i4,Rev-erb\alpha}}}{\left( {KI}_{4,Rev-erb\alpha}^{n_{i4,Rev-erb\alpha}}+x_{20}^{n_{i4,Rev-erb\alpha}} \right)}-k_{m,Rev-erb\alpha}x_{5}$$

$$\frac{dx_{6}}{dt}=u_{6}*\left( V_{0,Clk}+V_{1,Clk}\frac{x_{16}^{n_{a1,Clk}}}{\left( {KA}_{1,Clk}^{n_{a1,Clk}}+x_{16}^{n_{a1,Clk}} \right)} \right)*\frac{{KI}_{1,Clk}^{n_{i1,Clk}}}{\left( {KI}_{1,Clk}^{n_{i1,Clk}}+x_{13}^{n_{i1,Clk}} \right)}-k_{m,Clk}x_{6}$$

$$\frac{dx_{7}}{dt}=u_{7}*\left( V_{0,Bmal1}+V_{1,Bmal1}\frac{x_{16}^{n_{a1,Bmal1}}}{\left( {KA}_{1,Bmal1}^{n_{a1,Bmal1}}+x_{16}^{n_{a1,Bmal1}} \right)} \right)*\frac{{KI}_{1,Bmal1}^{n_{i1,Bmal1}}}{\left( {KI}_{1,Bmal1}^{n_{i1,Bmal1}}+x_{13}^{n_{i1,Bmal1}} \right)}-k_{m,Bmal1}x_{7}$$

$$\frac{dx_{8}}{dt}=u_{8}*\left( V_{0,Rorc}+V_{1,Rorc}\frac{x_{21}^{n_{a1,Rorc}}}{\left( {KA}_{1,Rorc}^{n_{a1,Rorc}}+x_{21}^{n_{a1,Rorc}} \right)}+V_{2,Rorc}\frac{x_{16}^{n_{a2,Rorc}}}{\left( {KA}_{2,Rorc}^{n_{a2,Rorc}}+x_{16}^{n_{a2,Rorc}} \right)} \right)*\frac{{KI}_{1,Rorc}^{n_{i1,Rorc}}}{\left( {KI}_{1,Rorc}^{n_{i1,Rorc}}+{x_{17}}^{{KI}_{1,Rorc}^{n_{i1,Rorc}}} \right)}*\frac{{KI}_{2,Rorc}^{n_{i2,Rorc}}}{\left( {KI}_{2,Rorc}^{n_{i2,Rorc}}+{x_{19}}^{n_{i2,Rorc}} \right)}*\frac{{KI}_{3,Rorc}^{n_{i3,Rorc}}}{\left( {KI}_{3,Rorc}^{n_{i3,Rorc}}+x_{18}^{n_{i3,Rorc}} \right)}*\frac{{KI}_{4,Rorc}^{n_{i4,Rorc}}}{\left( {KI}_{4,Rorc}^{n_{i4,Rorc}}+x_{20}^{n_{i4,Rorc}} \right)}*\frac{{KI}_{5,Rorc}^{n_{i5,Rorc}}}{\left( {KI}_{5,Rorc}^{n_{i5,Rorc}}+x_{13}^{n_{i5,Rorc}} \right)}-k_{m,Rorc}x_{8}$$

$$\frac{dx_{9}}{dt}=u_{9}*(t_{Per1}x_{1}-a_{PER1,CRY1}x_{9}x_{11}-a_{PER1,CRY2}x_{9}x_{12}+d_{PER1/CRY1}x_{17}+d_{PER1/CRY2}x_{19})-k_{p,PER1}x_{9}$$

$$\frac{dx_{10}}{dt}=u_{10}*{(t}_{Per2}x_{2}-a_{PER2,CRY1}x_{10}x_{11}-a_{PER2,CRY2}x_{10}x_{12}+d_{PER2/CRY1}x_{18}+d_{PER2/CRY2}x_{20})-k_{p,PER2}x_{10}$$

$$\frac{dx_{11}}{dt}=u_{11}*(t_{Cry1}x_{3}-a_{PER1,CRY1}x_{9}x_{11}-a_{PER2,CRY1}x_{10}x_{11}+d_{PER1/CRY1}x_{17}+d_{PER2/CRY1}x_{18})-k_{p,CRY1}x_{11}$$

$$\frac{dx_{12}}{dt}=u_{12}*(t_{Cry2}x_{4}-a_{PER1,CRY2}x_{9}x_{12}-a_{PER2,CRY2}x_{10}x_{12}+d_{PER1/CRY2}x_{19}+d_{PER2/CRY2}x_{20})-k_{p,CRY2}x_{12}$$

$$\frac{dx_{13}}{dt}=u_{13}*t_{Rev-erb\alpha}x_{5}-k_{p,PEV-ERB\alpha}x_{13}$$

$$\frac{dx_{14}}{dt}=u_{14}*(t_{Clk}x_{6}-a_{CLK,BMAL1}x_{14}x_{15}+d_{CLK/BMAL1}x_{21})-k_{p,CLK}x_{14}$$

$$\frac{dx_{15}}{dt}=u_{15}*(t_{Bmal1}x_{7}-a_{CLK,BMAL1}x_{14}x_{15}+d_{CLK/BMAL1}x_{21})-k_{p,BMAL1}x_{15}$$

$$\frac{dx_{16}}{dt}=u_{16}*t_{Rorc}x_{8}-k_{p,RORc}x_{16}$$

$$\frac{dx_{17}}{dt}={u_{17}*a}_{PER1,CRY1}x_{9}x_{11}-d_{PER1/CRY1}x_{17}$$

$$\frac{dx_{18}}{dt}=u_{18}*a_{PER2,CRY1}x_{10}x_{11}-d_{PER2/CRY1}x_{18}$$

$$\frac{dx_{19}}{dt}={u_{19}*a}_{PER1,CRY2}x_{9}x_{12}-d_{PER1/CRY2}x_{19}$$

$$\frac{dx_{20}}{dt}=u_{20}*a_{PER2,CRY2}x_{10}x_{12}-d_{PER2/CRY2}x_{20}$$

$$\frac{dx_{21}}{dt}={u_{21}*a}_{CLK,BMAL1}x_{14}x_{15}-d_{CLK/BMAL1}x_{21}$$

Table S3. Corresponding components of 21 state variables in mammalian circadian system.

| $x_{1}=Per1$ | $x_{2}=Per2$ |
| --- | --- |
| $x_{3}=Cry1$ | $x_{4}=Cry2$ |
| $x_{5}=Rev-erb\alpha$ | $x_{6}=Clk$ |
| $x_{7}=Bmal1$ | $x_{8}=Rorc$ |
| $x_{9}=PER1$ | $x_{10}=PER2$ |
| $x_{11}=CRY1$ | $x_{12}=CRY2$ |
| $x_{13}=REV-ERB\alpha$ | $x_{14}=CLK$ |
| $x_{15}=BMAL1$ | $x_{16}=RORc$ |
| $x_{17}=PER1/CRY1$ | $x_{18}=PER2/CRY1$ |
| $x_{19}=PER1/CRY2$ | $x_{20}=PER2/CRY2$ |
| $x_{21}=CLK/BMAL1$ |  |

Table S4. Parameters in mammalian circadian rhythm system.

| $V_{0,Per1}$=0.000001 | $V_{1,Per1}$=3 | $V_{0,Per2}$=0.09 |
| --- | --- | --- |
| $V_{1,Per2}$=3.29 | $V_{0,Cry1}$=0.26 | $V_{1,Cry1}$=2.44 |
| $V_{2,Cry1}$=2.89 | $V_{0,Cry2}$=1.29 | $V_{1,Cry2}$=2.72 |
| $V_{2,Cry2}$=0.1 | $V_{1,Rev-erb\alpha}$=11.06 | $V_{0,Clk}$=3.98 |
| $V_{1,Clk}$=3.36 | $V_{0,Bmal1}$=1.98 | $V_{1,Bmal1}$=4.12 |
| $V_{0,Rorc}$=0.06 | $V_{1,Rorc}$=3.55 | $V_{2,Rorc}$=0.46 |
| $n_{a1,Per1}$=2.0 | $n_{i1,Per1}$=2.0 | $n_{i2,Per1}$=1.0 |
| $n_{i3,Per1}$=2.0 | $n_{i4,Per1}$=4.0 | $n_{a1,Per2}$=10.0 |
| $n_{i1,Per2}$=1.0 | $n_{i2,Per2}$=1.0 | $n_{i3,Per2}$=9.0 |
| $n_{i4,Per2}$=8.0 | $n_{a1,Cry1}$ =4.91 | $n_{a2,Cry1}$=3.01 |
| $n_{i1,Cry1}$=1.0 | $n_{i2,Cry1}$=1.0 | $n_{i3,Cry1}$=6.0 |
| $n_{i4,Cry1}$=4.0 | $n_{i5,Cry1}$=2.24 | $n_{a1,Cry2}$=4.39 |
| $n_{a2,Cry2}$=4.43 | $n_{i1,Cry2}$=1.0 | $n_{i2,Cry2}$=1.0 |
| $n_{i3,Cry2}$=4.0 | $n_{i4,Cry2}$=8.0 | $n_{i5,Cry2}$=1.75 |
| $n_{a1,Rev-erb\alpha}$=4.40 | $n_{i1,Rev-erb\alpha}$=0.15 | $n_{i2,Rev-erb\alpha}$ =0.3 |
| $n_{i3,Rev-erb\alpha}$=7.0 | $n_{i4,Rev-erb\alpha}$=7.0 | $n_{a1,Clk}$=3.50 |
| $n_{i1,Clk}$=1.96 | $n_{a1,Bmal1}$=4.13 | $n_{i1,Bmal1}$=0.02 |
| $n_{a1,Rorc}$=1.57 | $n_{a2,Rorc}$=0.56 | $n_{i1,Rorc}$=1.0 |
| $n_{i2,Rorc}$=1.0 | $n_{i3,Rorc}$=7.0 | $n_{i4,Rorc}$=7.0 |
| $n_{i5,Rorc}$=4.33 | ${KA}_{1,Per1}$=1.98 | ${KI}_{1,Per1}$=1.07 |
| ${KI}_{2,Per1}$=3.96 | ${KI}_{3,Per1}$=1.68 | ${KI}_{4,Per1}$=3.11 |
| ${KA}_{1,Per2}=$1.90 | ${KI}_{1,Per2}$*=*4.51 | ${KI}_{2,Per2}$=2.98 |
| ${KI}_{3,Per2}$*=*2.24 | ${KI}_{4,Per2}$*=*3.31 | ${KA}_{1,Cry1}$*=*1.46 |

(Continued)

| ${KA}_{2,Cry1}$=3.76 | ${KI}_{1,Cry1}$=0.03 | ${KI}_{2,Cry1}$=0.77 |
| --- | --- | --- |
| ${KI}_{3,Cry1}$=3.59 | ${KI}_{4,Cry1}$*=*3.44 | ${KI}_{5,Cry1}$=2.82 |
| ${KA}_{1,Cry2}$=0.69 | ${KA}_{2,Cry2}$*=*2.96 | ${KI}_{1,Cry2}$*=*4.63 |
| ${KI}_{2,Cry2}$=2.95 | ${KI}_{3,Cry2}$=3.57 | ${KI}_{4,Cry2}$*=*2.75 |
| ${KI}_{5,Cry2}$*=*3.97 | ${KA}_{1,Rev-erb\alpha}$=3.15 | ${KI}_{1,Rev-erb\alpha}$*=*3.56 |
| ${KI}_{2,Rev-erb\alpha}$=3.62 | ${KI}_{3,Rev-erb\alpha}$=4.71 | ${KI}_{4,Rev-erb\alpha}$=1.23 |
| ${KA}_{1,Clk}$*=*1.59 | ${KI}_{1,Clk}$=0.83 | ${KA}_{1,Bmal1}$*=*2.59 |
| ${KI}_{1,Bmal1}$=2.47 | ${KA}_{1,Rorc}$=4.30 | ${KA}_{2,Rorc}$=4.89 |
| ${KI}_{1,Rorc}$*=*3.49 | ${KI}_{2,Rorc}$*=*2.34 | ${KI}_{3,Rorc}$*=*2.71 |
| ${KI}_{4,Rorc}$*=*2.09 | ${KI}_{5,Rorc}$*=*3.36 | $k_{m,Per1}$*=*2.18 |
| $k_{m,Per2}$*=*0.20 | $k_{m,Cry1}$*=*0.22 | $k_{m,Cry2}$*=*0.41 |
| $k_{m,Rev-erb\alpha}$=0.60 | $k_{m,Clk}$*=*3.19 | $k_{m,Bmal1}$*=*1.42 |
| $k_{m,Rorc}$*=*1.50 | $t_{Per1}$*=*3.05 | $t_{Per2}$=2.38 |
| $t_{Cry1}$*=*3.94 | $t_{Cry2}$*=*1.69 | $t_{Rev-erb\alpha}$=1.60 |
| $t_{Clk}$*=*3.04 | $t_{Bmal1}$*=*4.00 | $t_{Rorc}$*=*1.39 |
| $k_{p,PER1}$=2.58 | $k_{p,PER2}$=3.0 | $k_{p,CRY1}$=0.312 |
| $k_{p,CRY2}$=5.9 | $k_{p,REV-ERB\alpha}$=0.31 | $k_{p,CLK}$=1.52 |
| $k_{p,BMAL1}$=2.28 | $k_{p,RORc}$=3.33 | $a_{PER1,CRY1}$=3.57 |
| $a_{PER1,CRY2}$=3.12 | $a_{PER2,CRY1}$=3.81 | $a_{PER2,CRY2}$=4.0 |
| $a_{CLK,BMAL1}$=1.98 | $d_{PER1/CRY1}$=1.32 | $d_{PER1/CRY2}$=1.85 |
| $d_{PER2/CRY1}$=1.37 | $d_{PER2/CRY2}$=2.42 | $d_{CLK/BMAL1}$=0.97 |
| Control variables *u*_i_=1.0 (i=1, 2, … , 21) | | |

The original model equations of mammalian circadian rhythm system and physical meaning of all these parameters can be referred to the work of Mirsky et al. [1].

**2. Model equations for gastric cancer network**

$$\frac{dx_{1}}{dt}=u_{1}*\frac{1}{(1+a_{1}x_{2}^{n_{1}}+a_{2}x_{3}^{n_{2}}+a_{3}x_{9}^{n_{3}})}-x_{1}$$

$$\frac{dx_{2}}{dt}=u_{2}*\frac{a_{4}x_{4}^{n_{4}}+a_{5}x_{31}^{n_{5}}+a_{6}x_{33}^{n_{6}}+a_{7}x_{21}^{n_{7}}+a_{8}x_{22}^{n_{8}}+a_{9}x_{48}^{n_{9}}}{1+a_{4}x_{4}^{n_{4}}+a_{5}x_{31}^{n_{5}}+a_{6}x_{33}^{n_{6}}+a_{7}x_{21}^{n_{7}}+a_{8}x_{22}^{n_{8}}+a_{9}x_{48}^{n_{9}}}*\frac{1}{1+a_{10}x_{6}^{n_{10}}+a_{11}x_{44}^{n_{11}}+a_{12}x_{46}^{n_{12}}}-x_{2}$$

$\frac{dx_{3}}{dt}=u_{3}*\frac{a_{13}x_{4}^{n_{13}}+a_{14}x_{5}^{n_{14}}}{1+a_{13}x_{4}^{n_{13}}+a_{14}x_{5}^{n_{14}}}*\frac{1}{1+a_{15}x_{6}^{n_{15}}+a_{16}x_{7}^{n_{16}}}-x_{3}$

$$\frac{dx_{4}}{dt}=u_{4}*\frac{a_{17}x_{5}^{n_{17}}+a_{18}x_{19}^{n_{18}}+a_{19}x_{21}^{n_{19}}+a_{20}x_{22}^{n_{20}}+a_{21}x_{31}^{n_{21}}+a_{22}x_{33}^{n_{22}}+a_{23}x_{32}^{n_{23}}+a_{24}x_{48}^{n_{24}}}{1+a_{17}x_{5}^{n_{17}}+a_{18}x_{19}^{n_{18}}+a_{19}x_{21}^{n_{19}}+a_{20}x_{22}^{n_{20}}+a_{21}x_{31}^{n_{21}}+a_{22}x_{33}^{n_{22}}+a_{23}x_{32}^{n_{23}}+a_{24}x_{48}^{n_{24}}}*\frac{1}{1+a_{25}x_{8}^{n_{25}}+a_{26}x_{41}^{n_{26}}+a_{27}x_{42}^{n_{27}}+a_{28}x_{6}^{n_{28}}}-x_{4}$$

$$\frac{dx_{5}}{dt}=u_{5}*\frac{a_{29}x_{5}^{n_{29}}+a_{30}x_{4}^{n_{30}}}{1+a_{29}x_{5}^{n_{29}}+a_{30}x_{4}^{n_{30}}}*\frac{1}{1+a_{31}x_{1}^{n_{31}}+a_{32}x_{6}^{n_{32}}}-x_{5}$$

$$\frac{dx_{6}}{dt}=u_{6}*\frac{a_{33}x_{8}^{n_{33}}+a_{34}x_{41}^{n_{34}}+a_{35}x_{5}^{n_{35}}+a_{36}x_{42}^{n_{36}}+a_{37}x_{32}^{n_{37}}+a_{38}x_{21}^{n_{38}}+a_{39}x_{47}^{n_{39}}}{1+a_{33}x_{8}^{n_{33}}+a_{34}x_{41}^{n_{34}}+a_{35}x_{5}^{n_{35}}+a_{36}x_{42}^{n_{36}}+a_{37}x_{32}^{n_{37}}+a_{38}x_{21}^{n_{38}}+a_{39}x_{47}^{n_{39}}}*\frac{1}{1+a_{40}x_{4}^{n_{40}}+a_{41}x_{19}^{n_{41}}}-x_{6}$$

$$\frac{dx_{7}}{dt}=u_{7}*\frac{a_{42}x_{29}^{n_{42}}+a_{43}x_{41}^{n_{43}}+a_{44}x_{42}^{n_{44}}+a_{45}x_{20}^{n_{45}}+a_{46}x_{46}^{n_{46}}}{1+a_{42}x_{29}^{n_{42}}+a_{43}x_{41}^{n_{43}}+a_{44}x_{42}^{n_{44}}+a_{45}x_{20}^{n_{45}}+a_{46}x_{46}^{n_{46}}}*\frac{1}{1+a_{47}x_{4}^{n_{47}}+a_{48}x_{19}^{n_{48}}+a_{49}x_{3}^{n_{49}}+a_{50}x_{21}^{n_{50}}}-x_{7}$$

$$\frac{dx_{8}}{dt}=u_{8}*\frac{a_{51}x_{5}^{n_{51}}+a_{52}x_{4}^{n_{52}}+a_{53}x_{33}^{n_{53}}+a_{54}x_{10}^{n_{54}}}{1+a_{51}x_{5}^{n_{51}}+a_{52}x_{4}^{n_{52}}+a_{53}x_{33}^{n_{53}}+a_{54}x_{10}^{n_{54}}}*\frac{1}{1+a_{55}x_{19}^{n_{55}}}-x_{8}$$

$$\frac{dx_{9}}{dt}=u_{9}*\frac{a_{56}x_{10}^{n_{56}}+a_{57}x_{11}^{n_{57}}}{1+a_{56}x_{10}^{n_{56}}+a_{57}x_{11}^{n_{57}}}*\frac{1}{1+a_{58}x_{12}^{n_{58}}+a_{59}x_{6}^{n_{59}}}-x_{9}$$

$$\frac{dx_{10}}{dt}=u_{10}*\frac{a_{60}x_{16}^{n_{60}}+a_{61}x_{17}^{n_{61}}+a_{62}x_{15}^{n_{62}}+a_{63}x_{5}^{n_{63}}}{1+a_{60}x_{16}^{n_{60}}+a_{61}x_{17}^{n_{61}}+a_{62}x_{15}^{n_{62}}+a_{63}x_{5}^{n_{63}}}*\frac{1}{1+a_{64}x_{19}^{n_{64}}+a_{65}x_{12}^{n_{65}}}-x_{10}$$

$$\frac{dx_{11}}{dt}=u_{11}*\frac{a_{66}x_{39}^{n_{66}}+a_{67}x_{35}^{n_{67}}+a_{68}x_{37}^{n_{68}}}{1+a_{66}x_{39}^{n_{66}}+a_{67}x_{35}^{n_{67}}+a_{68}x_{37}^{n_{68}}}-x_{11}$$

$$\frac{dx_{12}}{dt}=u_{12}*\frac{a_{69}x_{19}^{n_{69}}+a_{70}x_{33}^{n_{70}}}{1+a_{69}x_{19}^{n_{69}}+a_{70}x_{33}^{n_{70}}}*\frac{1}{1+a_{71}x_{10}^{n_{71}}}-x_{12}$$

$$\frac{dx_{13}}{dt}=u_{13}*\frac{a_{72}x_{24}^{n_{72}}+a_{73}x_{33}^{n_{73}}+a_{74}x_{21}^{n_{74}}+a_{75}x_{48}^{n_{75}}}{1+a_{72}x_{24}^{n_{72}}+a_{73}x_{33}^{n_{73}}+a_{74}x_{21}^{n_{74}}+a_{75}x_{48}^{n_{75}}}*\frac{1}{1+a_{76}x_{8}^{n_{76}}+a_{77}x_{41}^{n_{77}}+a_{78}x_{16}^{n_{78}}+a_{79}x_{4}^{n_{79}}}-x_{13}$$

$$\frac{dx_{14}}{dt}=u_{14}*\frac{a_{80}x_{25}^{n_{80}}+a_{81}x_{26}^{n_{81}}+a_{82}x_{33}^{n_{82}}}{1+a_{80}x_{25}^{n_{80}}+a_{81}x_{26}^{n_{81}}+a_{82}x_{33}^{n_{82}}}*\frac{1}{1+a_{83}x_{16}^{83}+a_{84}x_{4}^{n_{84}}}-x_{14}$$

$$\frac{dx_{15}}{dt}=u_{15}*\frac{a_{85}x_{11}^{n_{85}}}{1+a_{85}x_{11}^{n_{85}}}-x_{15}$$

$$\frac{dx_{16}}{dt}=u_{16}*\frac{a_{86}x_{8}^{n_{86}}}{1+a_{86}x_{8}^{n_{86}}}*\frac{1}{1+a_{87}x_{19}^{n_{87}}+a_{88}x_{21}^{n_{88}}}-x_{16}$$

$$\frac{dx_{17}}{dt}=u_{17}*\frac{a_{89}x_{4}^{n_{89}}+a_{90}x_{8}^{n_{90}}}{1+a_{89}x_{4}^{n_{89}}+a_{90}x_{8}^{n_{90}}}*\frac{1}{1+a_{91}x_{13}^{n_{91}}+a_{92}x_{14}^{n_{92}}}-x_{17}$$

$$\frac{dx_{18}}{dt}=u_{18}*\frac{a_{93}x_{27}^{n_{93}}+a_{94}x_{25}^{n_{94}}+a_{95}x_{24}^{n_{95}}+a_{96}x_{28}^{n_{96}}}{1+a_{93}x_{27}^{n_{93}}+a_{94}x_{25}^{n_{94}}+a_{95}x_{24}^{n_{95}}+a_{96}x_{28}^{n_{96}}}-x_{18}$$

$$\frac{dx_{19}}{dt}=u_{19}*\frac{a_{97}x_{38}^{n_{97}}+a_{98}x_{18}^{n_{98}}+a_{99}x_{26}^{n_{99}}+a_{100}x_{29}^{n_{100}}+a_{101}x_{28}^{n_{101}}+a_{102}x_{27}^{n_{102}}+a_{103}x_{24}^{n_{103}}+a_{104}x_{25}^{n_{104}}+a_{105}x_{41}^{n_{105}}}{1+a_{97}x_{38}^{n_{97}}+a_{98}x_{18}^{n_{98}}+a_{99}x_{26}^{n_{99}}+a_{100}x_{29}^{n_{100}}+a_{101}x_{28}^{n_{101}}+a_{102}x_{27}^{n_{102}}+a_{103}x_{24}^{n_{103}}+a_{104}x_{25}^{n_{104}}+a_{105}x_{41}^{n_{105}}}*\frac{1}{1+a_{106}x_{20}^{n_{106}}}-x_{19}$$

$$\frac{dx_{20}}{dt}=u_{20}*\frac{1}{1+a_{107}x_{19}^{n_{107}}+a_{108}x_{33}^{n_{108}}+a_{109}x_{31}^{n_{109}}}-x_{20}$$

$$\frac{dx_{21}}{dt}=u_{21}*\frac{a_{110}x_{18}^{n_{110}}+a_{111}x_{28}^{n_{111}}+a_{112}x_{43}^{n_{112}}+a_{113}x_{45}^{n_{113}}}{1+a_{110}x_{18}^{n_{110}}+a_{111}x_{28}^{n_{111}}+a_{112}x_{43}^{n_{112}}+a_{113}x_{45}^{n_{113}}}*\frac{1}{1+a_{114}x_{20}^{n_{114}}+a_{115}x_{23}^{n_{115}}}-x_{21}$$

$$\frac{dx_{22}}{dt}=u_{22}*\frac{a_{116}x_{41}^{n_{116}}+a_{117}x_{35}^{n_{117}}+a_{118}x_{37}^{n_{118}}+a_{119}x_{38}^{n_{119}}+a_{120}x_{24}^{n_{120}}+a_{121}x_{25}^{n_{121}}+a_{122}x_{45}^{n_{122}}}{1+a_{116}x_{41}^{n_{116}}+a_{117}x_{35}^{n_{117}}+a_{118}x_{37}^{n_{118}}+a_{119}x_{38}^{n_{119}}+a_{120}x_{24}^{n_{120}}+a_{121}x_{25}^{n_{121}}+a_{122}x_{45}^{n_{122}}}*\frac{1}{1+a_{123}x_{20}^{n_{123}}+a_{124}x_{23}^{n_{124}}}-x_{22}$$

$$\frac{dx_{23}}{dt}=u_{23}*\frac{a_{125}x_{21}^{n_{125}}+a_{126}x_{22}^{n_{126}}}{1+a_{125}x_{21}^{n_{125}}+a_{126}x_{22}^{n_{126}}}-x_{23}$$

$$\frac{dx_{24}}{dt}=u_{24}*\frac{a_{127}x_{38}^{n_{127}}+a_{128}x_{32}^{n_{128}}+a_{129}x_{19}^{n_{129}}+a_{130}x_{27}^{n_{130}}+a_{131}x_{40}^{n_{131}}+a_{132}x_{37}^{n_{132}}+a_{133}x_{28}^{n_{133}}+a_{134}x_{43}^{n_{134}}}{1+a_{127}x_{38}^{n_{127}}+a_{128}x_{32}^{n_{128}}+a_{129}x_{19}^{n_{129}}+a_{130}x_{27}^{n_{130}}+a_{131}x_{40}^{n_{131}}+a_{132}x_{37}^{n_{132}}+a_{133}x_{28}^{n_{133}}+a_{134}x_{43}^{n_{134}}}-x_{24}$$

$$\frac{dx_{25}}{dt}=u_{25}*\frac{a_{135}x_{38}^{n_{135}}+a_{136}x_{28}^{n_{136}}}{1+a_{135}x_{38}^{n_{135}}+a_{136}x_{28}^{n_{136}}}-x_{25}$$

$$\frac{dx_{26}}{dt}=u_{26}*\frac{a_{137}x_{32}^{n_{137}}}{1+a_{137}x_{32}^{n_{137}}}*\frac{1}{1+a_{138}x_{8}^{n_{138}}}-x_{26}$$

$$\frac{dx_{27}}{dt}=u_{27}*\frac{a_{139}x_{21}^{n_{139}}+a_{140}x_{32}^{n_{140}}+a_{141}x_{31}^{n_{141}}}{1+a_{139}x_{21}^{n_{139}}+a_{140}x_{32}^{n_{140}}+a_{141}x_{31}^{n_{141}}}-x_{27}$$

$$\frac{dx_{28}}{dt}=u_{28}*\frac{a_{142}x_{25}^{n_{142}}+a_{143}x_{27}^{n_{143}}}{1+a_{142}x_{25}^{n_{142}}+a_{143}x_{27}^{n_{143}}}*\frac{1}{1+a_{144}x_{20}^{n_{144}}}-x_{28}$$

$$\frac{dx_{29}}{dt}=u_{29}*\frac{1}{1+a_{145}x_{28}^{n_{145}}+a_{146}x_{37}^{n_{146}}+a_{147}x_{27}^{n_{147}}+a_{148}x_{30}^{n_{148}}+a_{149}x_{9}^{n_{149}}}-x_{29}$$

$$\frac{dx_{30}}{dt}=u_{30}*\frac{a_{150}x_{41}^{n_{150}}+a_{151}x_{33}^{n_{151}}}{1+a_{150}x_{41}^{n_{150}}+a_{151}x_{33}^{n_{151}}}*\frac{1}{1+a_{152}x_{8}^{n_{152}}}-x_{30}$$

$$\frac{dx_{31}}{dt}=u_{31}*\frac{a_{153}x_{45}^{n_{153}}+a_{154}x_{27}^{n_{154}}+a_{155}x_{25}^{n_{155}}+a_{156}x_{26}^{n_{156}}}{1+a_{153}x_{45}^{n_{153}}+a_{154}x_{27}^{n_{154}}+a_{155}x_{25}^{n_{155}}+a_{156}x_{26}^{n_{156}}}*\frac{1}{1+a_{157}x_{29}^{n_{157}}+a_{158}x_{44}^{n_{158}}+a_{159}x_{46}^{n_{159}}}-x_{31}$$

$$\frac{dx_{32}}{dt}=u_{32}*\frac{a_{160}x_{19}^{n_{160}}+a_{161}x_{33}^{n_{161}}+a_{162}x_{4}^{n_{162}}}{1+a_{160}x_{19}^{n_{160}}+a_{161}x_{33}^{n_{161}}+a_{162}x_{4}^{n_{162}}}*\frac{1}{1+a_{163}x_{8}^{n_{163}}}-x_{32}$$

$$\frac{dx_{33}}{dt}=u_{33}*\frac{a_{164}x_{22}^{n_{164}}}{1+a_{164}x_{22}^{n_{164}}}*\frac{1}{1+a_{165}x_{34}^{n_{165}}+a_{166}x_{8}^{n_{166}}}-x_{33}$$

$$\frac{dx_{34}}{dt}=u_{34}*\frac{a_{167}x_{33}^{n_{167}}+a_{168}x_{21}^{n_{168}}+a_{169}x_{22}^{n_{169}}}{1+a_{167}x_{33}^{n_{167}}+a_{168}x_{21}^{n_{168}}+a_{169}x_{22}^{n_{169}}}*\frac{1}{1+a_{170}x_{37}^{n_{170}}+a_{171}x_{19}^{n_{171}}+a_{172}x_{35}^{n_{172}}+a_{173}x_{39}^{n_{173}}+a_{174}x_{41}^{n_{174}}}-x_{34}$$

$$\frac{dx_{35}}{dt}=u_{35}*\frac{a_{175}x_{33}^{n_{175}}+a_{176}x_{21}^{n_{176}}+a_{177}x_{22}^{n_{177}}}{1+a_{175}x_{33}^{n_{175}}+a_{176}x_{21}^{n_{176}}+a_{177}x_{22}^{n_{177}}}*\frac{1}{1+a_{178}x_{36}^{n_{178}}}-x_{35}$$

$$\frac{dx_{36}}{dt}=u_{36}*\frac{a_{179}x_{35}^{n_{179}}+a_{180}x_{39}^{n_{180}}}{1+a_{179}x_{35}^{n_{179}}+a_{180}x_{39}^{n_{180}}}*\frac{1}{1+a_{181}x_{42}^{n_{181}}}-x_{36}$$

$$\frac{dx_{37}}{dt}=u_{37}*\frac{a_{182}x_{33}^{n_{182}}+a_{183}x_{22}^{n_{183}}}{1+a_{182}x_{33}^{n_{182}}+a_{183}x_{22}^{n_{183}}}*\frac{1}{1+a_{184}x_{37}^{n_{184}}}-x_{37}$$

$$\frac{dx_{38}}{dt}=u_{38}*\frac{a_{185}x_{33}^{n_{185}}+a_{186}x_{22}^{n_{186}}}{1+a_{185}x_{33}^{n_{185}}+a_{186}x_{22}^{n_{186}}}-x_{38}$$

$$\frac{dx_{39}}{dt}=u_{39}*\frac{a_{187}x_{8}^{n_{187}}+a_{188}x_{33}^{n_{188}}+a_{189}x_{42}^{n_{189}}}{1+a_{187}x_{8}^{n_{187}}+a_{188}x_{33}^{n_{188}}+a_{189}x_{42}^{n_{189}}}*\frac{1}{1+a_{190}x_{18}^{n_{190}}}-x_{39}$$

$$\frac{dx_{40}}{dt}=u_{40}*\frac{a_{191}x_{33}^{n_{191}}+a_{192}x_{22}^{n_{192}}}{1+a_{191}x_{33}^{n_{191}}+a_{192}x_{22}^{n_{192}}}-x_{40}$$

$$\frac{dx_{41}}{dt}=u_{41}*\frac{a_{193}x_{28}^{n_{193}}+a_{194}x_{32}^{n_{194}}}{1+a_{193}x_{28}^{n_{193}}+a_{194}x_{32}^{n_{194}}}*\frac{1}{1+a_{195}x_{33}^{n_{195}}}-x_{41}$$

$$\frac{dx_{42}}{dt}=u_{42}*\frac{a_{196}x_{37}^{n_{196}}}{1+a_{196}x_{37}^{n_{196}}}*\frac{1}{1+a_{197}x_{36}^{n_{197}}}-x_{42}$$

$$\frac{dx_{43}}{dt}=u_{43}*\frac{a_{198}x_{36}^{n_{198}}+a_{199}x_{45}^{n_{199}}+a_{200}x_{25}^{n_{200}}+a_{201}x_{27}^{n_{201}}}{1+a_{198}x_{36}^{n_{198}}+a_{199}x_{45}^{n_{199}}+a_{200}x_{25}^{n_{200}}+a_{201}x_{27}^{n_{201}}}*\frac{1}{1+a_{202}x_{41}^{n_{202}}}-x_{43}$$

$$\frac{dx_{44}}{dt}=u_{44}*\frac{1}{1+a_{203}x_{19}^{n_{203}}+a_{204}x_{33}^{n_{204}}}-x_{44}$$

$$\frac{dx_{45}}{dt}=u_{45}*\frac{a_{205}x_{31}^{n_{205}}}{1+a_{205}x_{31}^{n_{205}}}-x_{45}$$

$$\frac{dx_{46}}{dt}=u_{46}*\frac{a_{206}x_{46}^{n_{206}}+a_{207}x_{48}^{n_{207}}+a_{208}x_{43}^{n_{208}}}{1+a_{206}x_{46}^{n_{206}}+a_{207}x_{48}^{n_{207}}+a_{208}x_{43}^{n_{208}}}*\frac{1}{1+a_{209}x_{42}^{n_{209}}}-x_{46}$$

$$\frac{dx_{47}}{dt}=u_{47}*\frac{a_{210}x_{31}^{n_{210}}+a_{211}x_{47}^{n_{211}}}{1+a_{210}x_{31}^{n_{210}}+a_{211}x_{47}^{n_{211}}}*\frac{1}{1+a_{212}x_{46}^{n_{212}}}-x_{47}$$

$$\frac{dx_{48}}{dt}=u_{48}*\frac{a_{213}x_{46}^{n_{213}}}{1+a_{213}x_{46}^{n_{213}}}*\frac{1}{1+a_{214}x_{47}^{n_{214}}+a_{215}x_{44}^{n_{215}}}-x_{48}$$

Where *a*_i_=7, *n*_i_=3 (i=1, 2, … , 215); control variables *u*_j_=1.0 (j=1, 2, … , 48). The original model equations of gastric cancer network can be found in the reference [2].

Table S5. Corresponding components of 48 state variables in gastric cancer network.

| $x_{1}=Rb$ | $x_{2}=CyclinD/CDK4,6$ | $x_{3}=CyclinE/CDK2$ |
| --- | --- | --- |
| $x_{4}=c-Myc$ | $x_{5}=E2F$ | $x_{6}=p21$ |
| $x_{7}=p27$ | $x_{8}=p53$ | $x_{9}=Caspase3,7$ |
| $x_{10}=Cytochromec$ | $x_{11}=Caspase8,10$ | $x_{12}=XIAP$ |
| $x_{13}=Bcl-2$ | $x_{14}=Bcl-xL$ | $x_{15}=Bid$ |
| $x_{16}=BAD$ | $x_{17}=Bax$ | $x_{18}=Ras$ |
| $x_{19}=PI3K/Akt$ | $x_{20}=PTEN$ | $x_{21}=ERK$ |
| $x_{22}=JNK/p38$ | $x_{23}=MKP$ | $x_{24}=VEGF/VEGFR$ |
| $x_{25}=EGF/EGFR$ | $x_{26}=IGF/EGFR$ | $x_{27}=HGF/Met$ |
| $x_{28}=Integrin/FAK$ | $x_{29}=E-cadherin$ | $x_{30}=Zeb1/2$ |
| $x_{31}=\beta-catenin$ | $x_{32}=HIF$ | $x_{33}=NF-kB$ |
| $x_{34}=ikB$ | $x_{35}=TNF-\alpha$ | $x_{36}=IL-10$ |
| $x_{37}=IL-1$ | $x_{38}=IL-8$ | $x_{39}=Fas$ |
| $x_{40}=COX-2$ | $x_{41}=TGF-\beta$ | $x_{42}=IFN-\gamma$ |
| $x_{43}=STAT3$ | $x_{44}=GSK-3\beta$ | $x_{45}=Gastrin/CC2R$ |
| $x_{46}=Sox2$ | $x_{47}=Cdx2$ | $x_{48}=Shh$ |

References:

1. Mirsky HP, Liu AC, Welsh DK, Kay SA, Doyle FJ. 2009 A model of the cell-autonomous mammalian circadian clock. Proceedings of the National Academy of Sciences, 106, 11107-11112. (doi: 10.1073/pnas.0904837106)

2. Li S, Zhu X, Liu B, Wang G, Ao P. 2015 Endogenous molecular network reveals two mechanisms of heterogeneity within gastric cancer. Oncotarget, 6, 13607–27. (doi: 10.18632/oncotarget.3633)
