# Supplementary material for: Reconciling periodic rhythms of large-scale biological networks by optimal control
Source: R Soc Open Sci. 2020 Jan 8;7(1):191698. doi: 10.1098/rsos.191698 (PMC7029949; doi:10.1098/rsos.191698)
Supplement: Point attractors in gastric cancer network [file rsos191698supp4.docx]

**Reconciling periodic rhythms of large-scale biological networks by optimal control**

Supplementary Data 4

**Table S6. Corresponding state variables values of six point attractors**

| Components | POA1 | POA2 | POA3 | POA4 | POA5 | POA6 |
| --- | --- | --- | --- | --- | --- | --- |
| *x*_1_ | 1.0000 | 1.0000 | 1.0000 | 0.9409 | 0.2155 | 0.1395 |
| *x*_2_ | 0.0006 | 0.0000 | 0.0000 | 0.1587 | 0.8000 | 0.8041 |
| *x*_3_ | 0.0000 | 0.0000 | 0.0000 | 0.1641 | 0.1962 | 0.7116 |
| *x*_4_ | 0.0156 | 0.0098 | 0.0026 | 0.3064 | 0.3085 | 0.3072 |
| *x*_5_ | 0.0000 | 0.0000 | 0.0000 | 0.0245 | 0.1843 | 0.6998 |
| *x*_6_ | 0.0000 | 0.0000 | 0.7298 | 0.1207 | 0.1220 | 0.1237 |
| *x*_7_ | 0.9340 | 0.9233 | 0.9233 | 0.1097 | 0.0828 | 0.0639 |
| *x*_8_ | 0.0000 | 0.0000 | 0.0000 | 0.0514 | 0.0524 | 0.1019 |
| *x*_9_ | 0.0000 | 0.0000 | 0.0000 | 0.0825 | 0.0821 | 0.0823 |
| *x*_10_ | 0.0000 | 0.0000 | 0.0000 | 0.0346 | 0.0354 | 0.0633 |
| *x*_11_ | 0.0000 | 0.0000 | 0.0000 | 0.4939 | 0.4970 | 0.4960 |
| *x*_12_ | 0.0098 | 0.0098 | 0.0098 | 0.8654 | 0.8703 | 0.8688 |
| *x*_13_ | 0.0059 | 0.0000 | 0.0000 | 0.2846 | 0.2733 | 0.2707 |
| *x*_14_ | 0.0000 | 0.0000 | 0.0000 | 0.7448 | 0.7439 | 0.7443 |
| *x*_15_ | 0.0000 | 0.0000 | 0.0000 | 0.4576 | 0.4620 | 0.4606 |
| *x*_16_ | 0.0000 | 0.0000 | 0.0000 | 0.0001 | 0.0001 | 0.0009 |
| *x*_17_ | 0.0000 | 0.0000 | 0.0000 | 0.0415 | 0.0425 | 0.0432 |
| *x*_18_ | 0.0000 | 0.0000 | 0.0000 | 0.9507 | 0.9546 | 0.9546 |
| *x*_19_ | 0.1122 | 0.1122 | 0.1122 | 0.9537 | 0.9678 | 0.9678 |
| *x*_20_ | 0.9902 | 0.9902 | 0.9902 | 0.1337 | 0.0800 | 0.0796 |
| *x*_21_ | 0.0000 | 0.0000 | 0.0000 | 0.4331 | 0.4388 | 0.4388 |
| *x*_22_ | 0.0000 | 0.0000 | 0.0000 | 0.4416 | 0.4432 | 0.4432 |
| *x*_23_ | 0.0000 | 0.0000 | 0.0000 | 0.5395 | 0.5456 | 0.5456 |
| *x*_24_ | 0.0098 | 0.0098 | 0.0098 | 0.9560 | 0.9595 | 0.9593 |
| *x*_25_ | 0.0000 | 0.0000 | 0.0000 | 0.8483 | 0.8574 | 0.8572 |
| *x*_26_ | 0.0000 | 0.0000 | 0.0000 | 0.8201 | 0.8224 | 0.8143 |
| *x*_27_ | 0.0000 | 0.0000 | 0.0000 | 0.8384 | 0.9093 | 0.9091 |
| *x*_28_ | 0.0000 | 0.0000 | 0.0000 | 0.8789 | 0.9031 | 0.9031 |
| *x*_29_ | 1.0000 | 1.0000 | 1.0000 | 0.0790 | 0.0704 | 0.0706 |
| *x*_30_ | 0.0000 | 0.0000 | 0.0000 | 0.7033 | 0.7035 | 0.7006 |
| *x*_31_ | 0.0000 | 0.0000 | 0.0000 | 0.1746 | 0.8805 | 0.8843 |
| *x*_32_ | 0.0098 | 0.0098 | 0.0098 | 0.8683 | 0.8730 | 0.8673 |
| *x*_33_ | 0.0000 | 0.0000 | 0.0000 | 0.3751 | 0.3776 | 0.3753 |
| *x*_34_ | 0.0000 | 0.0000 | 0.0000 | 0.0603 | 0.0592 | 0.0590 |
| *x*_35_ | 0.0000 | 0.0000 | 0.0000 | 0.4483 | 0.4501 | 0.4497 |
| *x*_36_ | 0.0000 | 0.0000 | 0.0000 | 0.3694 | 0.3718 | 0.3714 |
| *x*_37_ | 0.0000 | 0.0000 | 0.0000 | 0.3666 | 0.3680 | 0.3673 |
| *x*_38_ | 0.0000 | 0.0000 | 0.0000 | 0.4930 | 0.4965 | 0.4948 |
| *x*_39_ | 0.0000 | 0.0000 | 0.0000 | 0.0420 | 0.0422 | 0.0421 |
| *x*_40_ | 0.0000 | 0.0000 | 0.0000 | 0.4930 | 0.4965 | 0.4948 |
| *x*_41_ | 0.0000 | 0.0000 | 0.0000 | 0.6595 | 0.6590 | 0.6619 |
| *x*_42_ | 0.0000 | 0.0000 | 0.0000 | 0.1895 | 0.1904 | 0.1896 |
| *x*_43_ | 0.0000 | 0.0000 | 0.0000 | 0.2983 | 0.3107 | 0.3081 |
| *x*_44_ | 0.9902 | 0.9902 | 0.9902 | 0.1344 | 0.1295 | 0.1296 |
| *x*_45_ | 0.0000 | 0.0000 | 0.0000 | 0.0359 | 0.8270 | 0.8288 |
| *x*_46_ | 0.7389 | 0.0000 | 0.0000 | 0.8485 | 0.2009 | 0.1949 |
| *x*_47_ | 0.0000 | 0.0000 | 0.7370 | 0.0068 | 0.8528 | 0.8581 |
| *x*_48_ | 0.0947 | 0.0000 | 0.0000 | 0.7969 | 0.0100 | 0.0091 |
